# Inequities in urban emergency medical services

**DOI:** 10.1038/s44401-026-00148-2

**Published:** 2026-09-14

**Authors:** Christin Salley, Neda Mohammadi, John E. Taylor, Joe F. Bozeman III

**Affiliations:** 1 School of Civil and Environmental Engineering, Georgia Institute of Technology, Atlanta, GA USA; 2 Department of Engineering Management and Systems Engineering, George Washington University, Washington, DC USA; 3 School of Project Management, The University of Sydney, Sydney, NSW Australia; 4 School of Public Policy, Georgia Institute of Technology, Atlanta, GA USA

**Keywords:** Health care, Medical research

## Abstract

In the United States, systems such as Emergency Medical Services (EMS) provide rapid aid and medical care during emergencies and crisis events. EMS operations purportedly treat all callers equally, noting that certain information, such as select demographics, remains veiled until on the scene. However, disparities still persist, highlighting the need for further research investigating whether and how pre-existing inequities affect EMS response efforts. Here, we investigated differences in response times in New York City through regression analyses to (1) examine statistical disparities and (2) discover potential barriers that may contribute to disparities identified. Using data from the Fire Department of New York City, we analyzed 15,349,368 EMS response times from 2010 to 2020 to examine disparity and 1,363,382 response times from 2020 to investigate potential barriers. Results revealed significant inconsistencies in response times and failure to meet industry standards in higher-risk areas. This study elucidates the need for addressing such inequities and advancing equitable emergency management practices.

## Introduction

In the United States (U.S.), Emergency Medical Services (EMS) at various levels operate under diverse protocols and regulations rather than a unified framework, resulting in a decentralized system where decisions surrounding emergency response are shaped by a variety of factors^1^. More specifically, EMS response begins at the stages of call taking and dispatch, where dispatchers must collect caller-specific information and make critical decisions based on the environmental and contextual details provided about the incident. These processes are further shaped by agency-specific protocols and pre-arrival instructions, which can vary substantially across jurisdictions. As a result, emergency response practices may differ widely based on factors such as geographic location, neighborhood infrastructure, funding availability, technological systems, and local governance structures, at times even complicating coordination between neighboring jurisdictions due to incompatible systems, differing operational priorities, or variations in resource capacity. Although EMS professionals receive specialized training to serve this critical public safety role, the operational variability inherent in this overall system (i.e., various CAD providers, home vs. state-mandated rules, etc.) reflects broader challenges in ensuring consistent emergency response outcomes for callers. While the EMS process is designed to provide a standardized caller experience and mitigate biased decision-making^2^, with demographics like race and age often remaining unknown until arrival on the scene^3^, this design does not inherently eliminate disparities that can arise. In practice, pre-existing social and health inequities can still be exacerbated when emergency services and response resources are not sufficiently equipped to recognize and address structural barriers such as language differences, variations in call type interpretation, transportation and road infrastructure limitations, or uneven resource availability. These factors can lead to delayed care, misallocation of resources, or unmet needs in historically underserved communities. Essentially, despite efforts to provide equal treatment in caller experience or intervention execution through established protocols and procedures, EMS systems may still experience and perpetuate inequities in emergency response across communities due to broader structurally patterned disparities, as equal dispatch procedures do not necessarily produce equal service outcomes.

The growing concerns around environmental injustices and the call for equity to be embedded in essential societal systems^4,5^ highlight the need for emergency response systems to better address systemic inequities and deliver more equitable care. This is evidenced by the passage of laws that help to address inequities such as the Justice40 Initiative, the Creating Helpful Incentives to Produce Semiconductors—CHIPS—and Science Act, the Inflation Reduction Act, and the 2022–2026 FEMA Strategic Plan (with equity in emergency management as its first goal)^6–11^. These federal initiatives had disaster or emergency factors associated with them but do not address the EMS matters previously discussed directly. In acknowledging these federal efforts and leveraging their momentum, it is crucial to examine the existing disparities observed in such emergency management systems to identify the specific barriers that can hinder progress.

Previous studies underscore that the detrimental effects from emergencies are not solely attributed to the harm caused by such events, but rather it is the convergence of these with pre-existing social inequalities that exacerbates the disparities already present in society^12^, further amplifying the notion that an emergency’s impacts are beyond what the hazard itself causes. For instance, as urbanization and population aging continue to increase, equitable access to healthcare services has emerged as a persistent spatial and infrastructural challenge, prompting the development of more innovative approaches for analyzing accessibility and spatial planning strategies in vulnerable communities disproportionately affected by these issues^13^. Studies also show, however, some of these increasingly data-driven approaches for reducing disparities, such as models addressing gender disparities in specific call types, may also introduce additional concerns surrounding algorithmic bias, including unfair performance or underperformance for historically disenfranchised groups^14^. Work performed in England outlines inequities that exist with certain populations, particularly when flooding events occur, noting that the elderly are receiving disproportionate coverage^15^. Other findings suggest that residents with cheaper, more affordable healthcare led to slower ambulance response times^16^. Furthermore, previous work on the U.S. national scale suggests that there are cardiac arrest disparities in EMS for low-income areas, where low-income residents are less likely to receive service that met industry standard times compared to their middle- and high-income counterparts^3^. Overall, while research on disparities in response times and subpopulations explicitly is still developing, recent studies have begun to uncover quantitative relationships between sociodemographic factors and emergency response times. Yet, there still exists a research gap in quantifying and examining disparities in emergency response efforts for all call types or incidents, specifically as it pertains to time taken to reach the incident scene versus actions undertaken while on scene or departing from the scene. Additionally, there is a paucity in research investigating U.S. EMS response times for same-system dispatches, given the significant variability that can occur. This gap contrasts with broader studies involving multiple protocols or international research with different governance, laws, and sociodemographics, which may affect the conclusions drawn or the applicability of the findings.

To address the research gaps previously mentioned and test if there are time disparities in a system designed for uniform service across all areas, as well as investigate potential barriers having a significant impact on disparities found in emergency response, our study investigated the following null hypotheses to test whether disparities exist and identify contributing factors:

- H1: There is no statistically significant difference in response times across sociodemographic areas within the same emergency services and dispatch system in a major metropolitan city.
- H2: The response barriers formulated have no statistically significant relationship with response time disparities identified.

This study accomplishes two key objectives. Firstly, it establishes and quantifies the presence of a disparity in general emergency response time across various call types and severity levels in a major U.S. city with the same dispatch system. Secondly, it formulates and tests the barriers contributing to disparities discovered to identify statistically significant emergency response time inequities that may exist. Collectively, these contributions have the potential to catalyze meaningful action to address inequities in emergency response efforts and drive progress toward more equitable emergency management practices by providing insight into contextual factors influencing emergency response disparities and supporting efforts to improve response equity.

## Results

### Analysis of New York City EMS—case study

To test our hypotheses, we conducted a case study. The focus of our study was the largest city in the U.S., New York City. New York City has publicly available data on EMS Incident Dispatch Data provided by the Fire Department of New York City (FDNY) that is de-identified (i.e., does not contain private health information) and includes information such as call type, severity level, dispatch time, travel time, response time, and location^17^. Given the nature of our analysis (further explained in the “Methods” section), the spatial scale of the study was set at the county level to facilitate comparisons among the recognized boroughs within New York City. Because borough and county boundaries align in New York City, we use these terms interchangeably throughout the study. The five boroughs of New York City are the Bronx (Bronx County), Brooklyn (Kings County), Manhattan (New York County), Queens (Queens County), and Staten Island (Richmond County). Although New York City boroughs are internally heterogeneous, this spatial level was used for more geographic coverage, larger sample sizes, data interpretation, and availability of data on the established barriers. This approach enabled a more comprehensive understanding of the patterns and variations within the study area. The temporal scale of testing response phase disparities was a decade. We analyzed EMS time values from 2010 to 2020, resulting in 15,349,368 data points. The temporal scale of a decade was found to be most suitable for our analysis, allowing for longitudinal observations and enhanced contextual understanding and reliability of the assessment performed. The temporal scale of testing barriers was the year 2020, resulting in 1,363,382 data points. The barrier analysis is restricted to 2020 because it requires sociodemographic variables from sources such as the U.S. Census/ACS, which are available at a single national reference year rather than annually. Furthermore, it is important to note that with this data, all reported times are with the same central dispatch system, adding consistency across the dataset in terms of reporting and protocols with EMS operations^18^.

In the U.S., key organizations that set industry standards for various phases of emergency response include organizations such as the Association of Public-Safety Communications Officials (APCO), the National Emergency Number Association (NENA), and the National Fire Protection Association (NFPA). APCO and NENA primarily focus on call-taking and processing, while NFPA explicitly outlines standards for response times. As the data is provided by a fire department and the study is analyzing response times, the performance objectives for EMS in this study were centered on those outlined by an NFPA standard, specifically NFPA 1710 “Standard for the Organization and Deployment of Fire Suppression Operations, Emergency Medical Operations, and Special Operations to the Public by Career Fire Departments”^19^. This document outlines industry standards for agencies identified by the Authority Having Jurisdiction (AHJ), like the FDNY. The definition for response time as defined by the EMS data descriptions^17^ and aligning with the NFPA standard is the following:

- Response Time (FDNY): “The time elapsed in seconds between the incident datetime and the first on scene datetime.”
  - NFPA 1710 standard:
    - Dispatch Time + Travel Time: 390 s (about 6 and a half minutes) to 420 s (about 7 min)

In the present study, we adopt the FDNY’s definition of response time. Supplementary Table 1 provides an overview of definitions from both the data descriptions and NFPA 1710, which set the threshold mentioned above.

#### Formulation of barriers

Despite substantial research on barriers in emergency management more broadly, there is a notable gap in studies specifically documenting barriers to emergency response times and their connection to disparities in EMS. By analyzing the cyclical nature and interrelationships among the four phases of emergency management, key themes of barriers affecting response efforts were identified through analyzing literature related to mitigation^20–23^, preparedness^20,24–29^, and recovery^30–32^. A set of response barriers was then developed, and corresponding variables were selected to represent each barrier through a combination of internal and external validation processes. Similar to Chowdhury and Zhu (2023)^33^, internal validation involved iterative review and discussion among the research team, while external validation consisted of refining the identified barriers through synthesis of the aforementioned literature pertaining to barriers across the other phases of emergency management. These barriers include: (1) Communication Systems, (2) Community Capacity, (3) Community Culture and Priorities, (4) Dependent Populations, (5) Incident Translation, (6) Medical System Capacity, and (7) Physical Infrastructure. Each barrier is defined in further detail in the “Methods” section (Table 1). Table 2 details each barrier, its associated indicators as identified by the research team, and the data used to represent them. If not addressed, these barriers could significantly impact the efficiency of emergency response times. The barriers identified in this study should not be viewed as hard category delineations, and future research could conduct sensitivity analyses to examine potential overlaps and collinearities across categories.

**Table 1:** Detailed description of barriers

Barrier	Description
Capacity (Community and Medical System)	Preparedness studies revealed that capacities of both communities and medical systems can affect response capabilities in terms of networks of support and insurance, which, if strong, can improve response resilience (Linnell, 2013; Ramsbottom et al., 2018) in addition to the number of facilities, organizations, or trained workers (Dunlop et al., 2016; Levi et al., 2011) to handle the demand for services.
Communication Systems	Previous studies on recovery have documented that telecommunication networks such as internet access or cellular connectivity are potential barriers to response when citizens do not have a connection (Rengaraju et al., 2021). Response personnel need to stay connected to communities to make announcements, disseminate information, and assess societal needs.
Community Culture and Priorities	Research on mitigation reported that there are value systems as it relates to health and social implications ingrained in a community that will shape decision-making and interaction with emergency management (Ramsbottom et al., 2018). Examples of this are how willing a person is to engage in community activities (Williams, 2004) or citizens taking responsibility for response efforts if they are less reliant on government agencies and more on local efforts (Sobelson et al., 2015).
Dependent Populations	Research on mitigation disclosed that properly supporting and aiding dependent populations, such as those who need additional assistance (e.g., young, old, those with disabilities, etc.), is another barrier to emergency management systems that can impede response efforts; this could be due to responders not having adequate resources needed to properly assist with this vulnerable population (Ton et al., 2019).
Incident Translation	Studies on preparedness demonstrated that incident translation or language (e.g., the caller’s ability to adequately describe the crisis event to the dispatcher) is a barrier (James et al., 2007). With this, it has been discovered certain populations are linguistically isolated and placed at a disadvantage when communicating with emergency operators (Nepal et al., 2012), as well as educational attainment attributing to the level of community engagement for disaster preparedness (Rivera, 2021).
Physical Infrastructure	Recovery research shows physical infrastructure such as transportation channels (i.e., traffic volume) has been found to impede response time (Gao and Cao, 2020) if there is congestion or a jam blocking optimal routes. Moreover, declining infrastructure due to historical environmental inequities, such as redlining and residential segregation in communities, leads to limited availability and accessibility of resources (Bozeman et al., 2023). These historical discriminatory practices still affect communities’ social and economic equity today.

**Table 2:** Corresponding data to barriers found impeding emergency response

Barrier	Indicator	Variable	Measure	Source
Communication Systems	Cellular Connectivity	Households with No Cellular Data Plan	Percent	American Community Survey (ACS)^a^
	Internet Access	Households with No Broadband of Any Type	Percent	American Community Survey (ACS)^a^
		Households with No Internet Access	Percent	American Community Survey (ACS)^a^
		Wi-Fi Hotspots	Count	Office of Technology and Innovation (OTI)^b^
Community Capacity	Networks of Support	Community Emergency Response Team (CERT) Program	Count	Local Emergency Management Agency^b^
		Community-based Organizations	Count	Local Service Agency^b^
		Readiness Events	Count	Local Emergency Management Agency^b^
	Insurance	Population with no Health Insurance Coverage	Percent	American Community Survey (ACS)^a^
Community Culture and Priorities	Social Implications	Income Inequality	Percent	American Community Survey (ACS)^a^
	Health Implications	Health Status (Mental and Physical)	Percent	CDC Places^c^
Dependent Populations	Additional Assistance	Population Between Ages 0-17 (i.e., Young Population)	Percent	American Community Survey (ACS)^a^
		Population Ages 65 and Over (i.e., Elderly)	Percent	American Community Survey (ACS)^a^
		Population with a Disability	Percent	American Community Survey (ACS)^a^
Incident Translation	Education	Population with less than High School Education (ages 25 and over)	Percent	American Community Survey (ACS)^a^
	Language	Households (interpreted as individuals) in Linguistic Isolation	Percent	EPA EJScreen^d^
Medical System Capacity	Number of Facilities	Firehouses	Count	FDNY^b^
Physical Infrastructure	Historical Implications	Residential Segregation	Percent	County Health Rankings^e^
	Traffic	Traffic Proximity and Volume	Percentile	EPA EJScreen^d^
	Roads	Street Closures	Count	Department of Transportation^b^

^a^AHRQ. (2020). *Social Determinants of Health Database*. AHRQ—Agency for Healthcare Research and Quality. https://www.ahrq.gov/sdoh/data-analytics/sdoh-data.html.

^b^City of New York. (2022). *Open Data for All New Yorkers*. NYC Open Data. https://opendata.cityofnewyork.us/.

^c^CDC. (2020). *Places: Local data for Better Health, county data 2020 release*. Centers for Disease Control and Prevention. https://chronicdata.cdc.gov/500-Cities-Places/PLACES-Local-Data-for-Better-Health-County-Data-20/dv4u-3x3q.

^d^EPA. (2020). EJScreen: Environmental Justice Screening and Mapping Tool Download EJScreen Data. EPA. https://www.epa.gov/ejscreen/download-ejscreen-data.

^e^County Health Rankings. (2020). Residential segregation—Black/White*. County Health Rankings & Roadmaps. https://www.countyhealthrankings.org/explore-health-rankings/county-health-rankings-model/health-factors/social-economic-factors/family-and-social-support/residential-segregation-blackwhite?year=2020&tab=1.

#### Disparities testing

Upon execution of an analysis of variance (ANOVA) on the five counties, we found that for response time (which includes both dispatch and travel time) all *p* values were <0.01, suggesting there are statistically significant differences among the counties and rejecting H1 (i.e., There is no statistically significant difference in response times across sociodemographic areas within the same emergency services and dispatch system in a major metropolitan city). To explore this further, Games-Howell post hoc tests for response time (see Table 3) revealed a deeper understanding. For this test, every possible combination of comparing county to county also rejected H1 with *p* values that were <0.01 as well. In examining the Games-Howell post hoc tests for response time, it was revealed that the Bronx experiences the slowest reported times on average, while the Richmond/Staten Island area experiences the fastest reported times on average. With this, the existence of a significant disparity is firmly established in an area that relies on an identical dispatch system and services for its daily EMS efforts. Additionally, the distribution of the data revealed to the research team a pronounced common characteristic or behavior across the distributions for each borough and each individual year in the time period of the present study, converging around the same point both on the *x*- and *y*-axes. Furthermore, the random-effects model reported a single effective size for response time of 0.0865, with a standard error of 0.0244. We elaborate on this latter point more in the “Discussion” section.

**Table 3:** Games-Howell post hoc test for response time

Group 1	Group 2	Mean Group 1	Mean Group 2	Difference	*p* value	Hedges	Reject null hypothesis 1?
BRONX	BROOKLYN	611.4412	542.7532	68.68802	<0.01	0.100105	TRUE
BRONX	MANHATTAN	611.4412	582.3069	29.13428	<0.01	0.038693	TRUE
BRONX	QUEENS	611.4412	507.4175	104.0237	<0.01	0.152029	TRUE
BRONX	RICHMOND/STATEN ISLAND	611.4412	454.9112	156.53	<0.01	0.21936	TRUE
BROOKLYN	MANHATTAN	542.7532	582.3069	-39.5537	<0.01	-0.056815	TRUE
BROOKLYN	QUEENS	542.7532	507.4175	35.33567	<0.01	0.056746	TRUE
BROOKLYN	RICHMOND/STATEN ISLAND	542.7532	454.9112	87.84198	<0.01	0.141628	TRUE
MANHATTAN	QUEENS	582.3069	507.4175	74.88941	<0.01	0.107532	TRUE
MANHATTAN	RICHMOND/STATEN ISLAND	582.3069	454.9112	127.3957	<0.01	0.174527	TRUE
QUEENS	RICHMOND/STATEN ISLAND	507.4175	454.9112	52.50631	<0.01	0.089248	TRUE

#### Industry standards

NFPA 1710 standards for response time should be completed between 390 s and 420 s. For New York City, over the course of a decade, the percentage of values meeting the threshold of 390 s was 41.98%, and the percentage of values meeting the threshold of 420 s was 47.75%. Although NFPA does not mandate a specific compliance rate in this standard, these reports highlight a critical gap: New York City falls short of this national benchmark in more than half of its EMS responses. Falling short of these benchmarks, even if they are aspirational, could mean the difference between life and death for individuals in emergency situations, once again underscoring the urgent need for strategies to close this gap in service disparity.

#### Barriers testing

We performed a Least Absolute Shrinkage and Selection Operator (LASSO) regression analysis to discover which formulated potential barriers were most strongly associated with the disparities observed. The LASSO regression resulted in four variables being noted as most influential in impeding emergency response efforts in New York City, forcing the rest of the variables to zero or insignificant values (see Table 4). The results of the LASSO rejected H2 (i.e., The response barriers formulated have no statistically significant relationship with response time disparities identified). The significant barriers were the Community Emergency Response Team (CERT) Program, Population with a Disability, Population with less than High School Education, and Traffic Proximity and Volume.

**Table 4:** Results of the LASSO regression, identifying the most relevant variables that have a significant impact on the dependent variable (i.e., reported response time)

Variable	Estimate	Standard error	*p* value
Community Emergency Response Team (CERT) Program	11.2	1.02	<0.0001
Population with a Disability	27.5	3.09	<0.0001
Population with less than High School Education	17.41	2.75	<0.0001
Traffic Proximity and Volume	14.39	1.13	<0.0001
Community-based Organizations	0	0	1
Firehouses	0	0	1
Health Status (Mental and Physical)	0	0	1
Households (interpreted as individuals) in Linguistic Isolation	0	0	1
Households with No Broadband of Any Type	0	0	1
Households with No Cellular Data Plan	0	0	1
Households with No Internet Access	0	0	1
Income Inequality	0	0	1
Population Ages 65 and Over (i.e., Elderly)	0	0	1
Population Between Ages 0-17 (i.e., Young Population)	0	0	1
Population with no Health Insurance Coverage	0	0	1
Readiness Events	0	0	1
Residential Segregation	0	0	1
Street Closures	0	0	1
Wi-Fi Hotspots	0	0	1

While correlation does not imply causation, the results suggest that addressing these four barriers could be pivotal for New York City in mitigating disparities during the response phase. It is important to note that these barriers are not the root “problem” per se (e.g., the presence of individuals with disabilities is not the issue; rather, the lack of appropriate resources or technology to adequately assist this group is the concern that needs addressing). These findings emphasize the pre-existing conditions in society (i.e., barriers) that currently can impact emergency response. Future policies and interventions can take insights from a study design like ours and take these factors into serious consideration to address and bridge the disparities observed in response times.

## Discussion

**Fig. 1:**
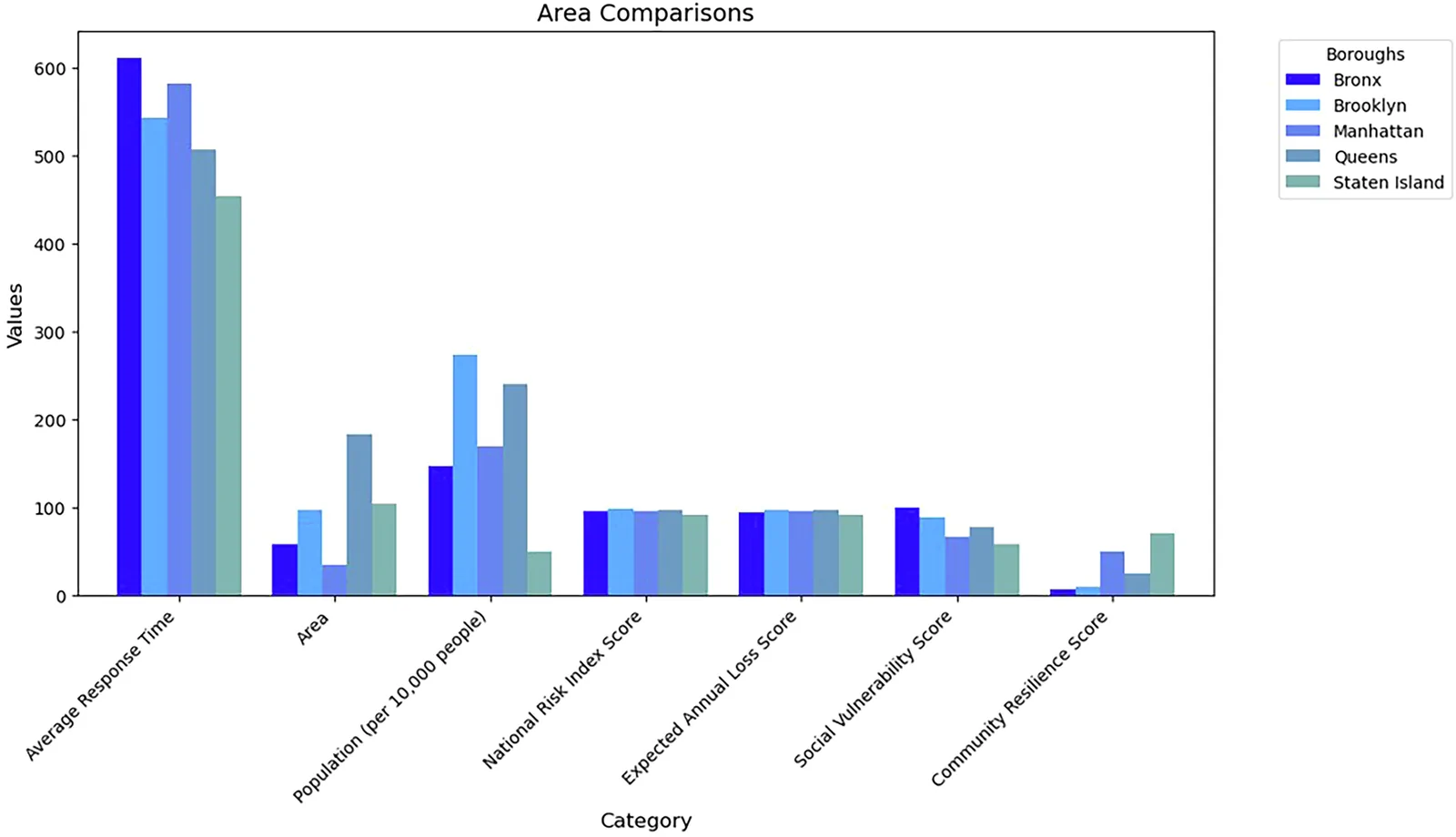
Bar chart comparison of each borough in relation to certain risk-based characteristics. The figure highlights that as social vulnerability increases and community resilience decreases, emergency response times tend to become slower.

Through performing ANOVA and Games-Howell post hoc tests on a decade-long dataset covering the five boroughs of New York City, this study reveals a statistically significant difference in average response times across these areas. However, given the exceptionally large sample size of observations, statistical significance should be interpreted with caution, as even relatively small differences are likely to produce highly significant *p* values. As such, the practical and operational implications of observed response-time differences, including absolute differences measured in seconds (i.e., the largest difference being 156.53 s between two counties) and associated effect sizes (i.e., 0.0865), are important considerations when interpreting these findings. The Bronx (Bronx County) experiences the slowest reported times on average, while Richmond/Staten Island (Richmond County) experiences the fastest reported times on average. This finding is particularly noteworthy when examined in the context of FEMA’s National Risk Index (NRI), which assesses an area’s risk based on expected annual loss, social vulnerability, and community resilience. Despite their higher NRI risk designation (“Relatively High”), the Bronx (Bronx County), Brooklyn (Kings County), Manhattan (New York County), and Queens (Queens County) all experience longer response times than Staten Island (Richmond County), which has the lowest NRI risk designation (“Relatively Moderate”)^34^. Figure 1 reinforces this pattern by showing a clear directional relationship: as social vulnerability increases and community resilience decreases, emergency response times become longer. Since FEMA administers programs and systems that provide aid pertaining to emergencies, these findings can inform regional preparedness by highlighting where response delays and social vulnerability intersect. Such insights can guide targeted resource allocation, prioritize funding for high-need areas in alignment with FEMA’s NRI and identified response-time inequities in this study, and support more equitable planning and capacity-building efforts across the region.

**Fig. 2:**
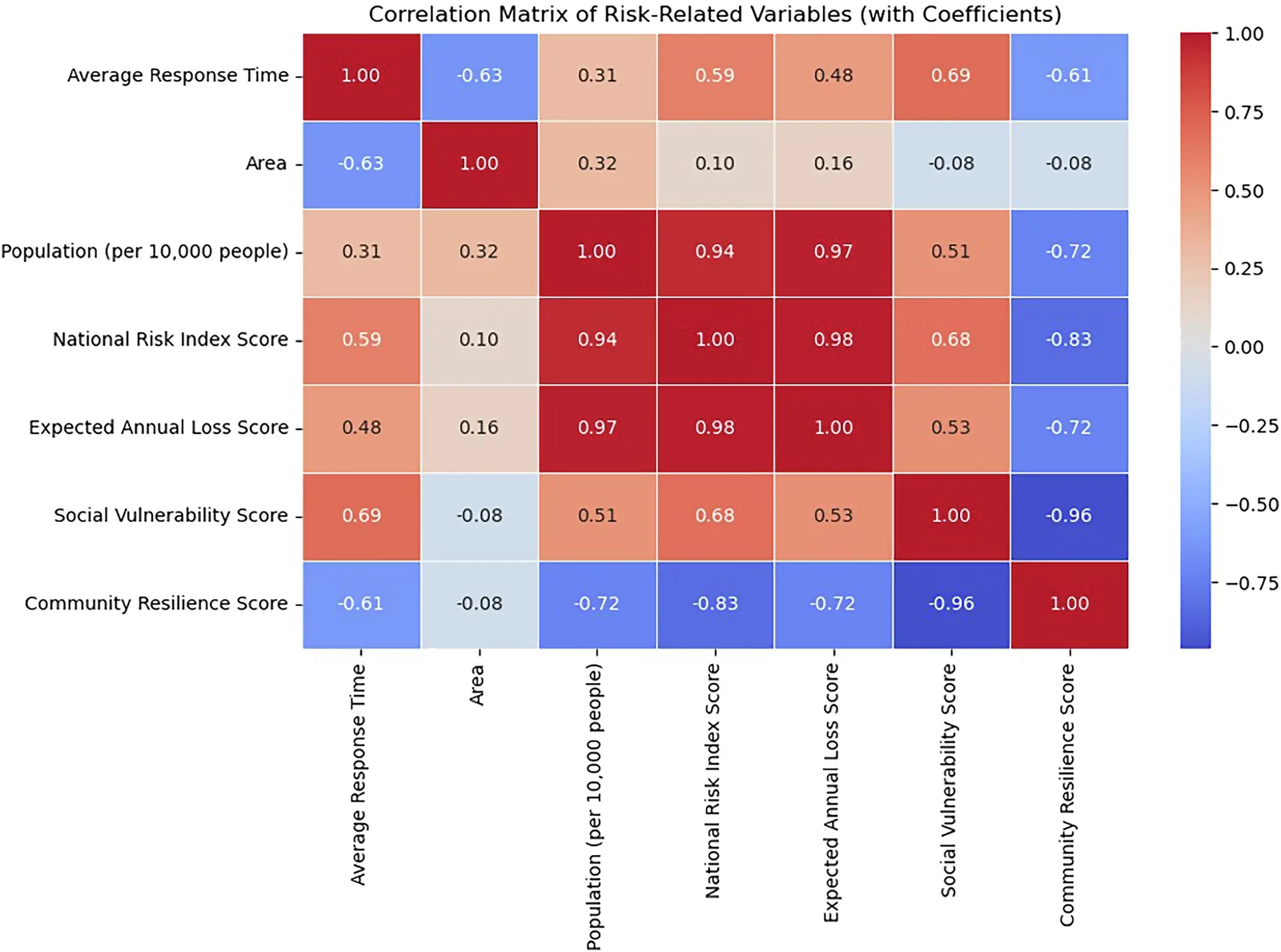
Correlations of risk-based characteristics (NRI elements) in comparison to average response time. The observed correlations suggest that emergency response disparities are associated not only with geographic conditions, but also with broader patterns of social vulnerability and community resilience.

Figure 2 provides further insights by revealing correlational relationships for response time and NRI elements. Area size is negatively correlated with response time (*r* = −0.63), suggesting that larger boroughs might experience slightly faster response times. This contradicts the expectation that greater distances inherently lead to longer wait times, indicating that infrastructure, dispatch logistics, or resource distribution may play a more dominant role than sheer geographic size. Population density is weakly correlated with response times (*r* = 0.31), contradicting the expectation that higher population density would lead to longer response times due to congestion or demand. This suggests that density may not be strong enough to be a primary determinant. Expected annual loss is weakly correlated with response times (*r* = 0.48), contradicting the expectation that financial disaster risk directly impacts response time efficiency. This suggests that while financial risk assessments are important for disaster preparedness, they do not appear to be a key factor influencing disparities in emergency response times. Higher social vulnerability scores are positively correlated with longer response times (*r* = 0.69), suggesting that boroughs with greater social vulnerability experience slower response times, reinforcing concerns about systemic inequities in emergency response. Community resilience is negatively correlated with response time (*r* = −0.61), meaning that boroughs with stronger resilience could receive faster responses, highlighting the role of preparedness, resources, and infrastructure in response efficiency.

These insights challenge assumptions that geographic constraints or financial risk alone drive response time disparities. Instead, the stronger correlation between response time and social vulnerability suggests that systemic factors, such as underinvestment in emergency services, uneven resource allocation, and historical disparities in infrastructure, are more critical determinants of response efficiency. These findings reinforce the notion that emergency response outcomes are shaped not only by hazard exposure or geographic characteristics, but also by broader social and infrastructural conditions influencing community resilience and service accessibility.

The practical and operational implications of observed response-time disparities are also important when considering time-sensitive medical emergencies. Results showed that New York City does not meet the standards outlined by NFPA. These disparities in response can have life-and-death implications. For a medical situation such as cardiac arrest, effects such as neurological damage can occur within 4–6 min after collapse without proper intervention^35^. Scrutinizing the results of this study, FDNY only meets this threshold (4–6 min) roughly 30% of the time. This means 1 in 3 people experiencing cardiac arrest and trying to access FDNY could end in neurological damage or fatality. This suggests that a substantial proportion of individuals experiencing cardiac arrest may face elevated risks of neurological damage or fatality due to delayed intervention, and areas with slower response times could endure even lower survival rates for such time-urgent conditions. Additionally, Hedges g and the meta-analysis conducted measured the magnitude of the response time interval. Research shows that “small” effect sizes can have large impacts on reducing death rates (e.g., suicide rates) at values such as 0.1^36^. Therefore, our study’s effect size of 0.0865 for response time should not be dismissed as insignificant. While it may be classified under the usual threshold for a small effect, it is crucial to consider the context of the effect being measured. Even relatively modest effect sizes can translate into substantial reductions in mortality rates with proper intervention, and can have profound implications for health, safety, and general well-being. In a city the size of New York City, this can mean hundreds or thousands of affected lives per year. Taken together, the present study’s effect size aligns with evidence that small effect sizes in human-centered research can have significant real-world impacts.

The four barriers discovered in the present study (i.e., CERT, Population with a Disability, Population with less than High School Education, and Traffic Proximity and Volume) exhibit a heightened level of coherence and relevance to the response disparities New York City experiences. Rather than representing problems in themselves, these barriers identify areas where New York City can begin targeted assessment to potentially strengthen response capabilities. With the first barrier, CERT, community ownership and involvement can be very beneficial for effective emergency management. When communities are involved in programs and initiatives for emergency management activities, it can lead to long-term, persistent alleviation of hazards and risks^37^. New York City is known to be the largest city in the U.S., and not having enough community assistance with emergency response programs, such as CERT, can pose a substantial problem when trying to execute equitable response and meet industry standards.

People with a disability (e.g., difficulties with vision, cognitive function, etc.) were identified as another significant barrier. Those with disabilities can be more difficult to locate during an emergency and harder to assess their needs^38^. Issues such as communicating enough information that properly allows dispatchers to assess the situation can occur if the person has a speech or hearing impairment, resulting in delayed response times. Nearly 1 million people in New York City have a reported disability^39^, but with proper mitigation and preparation for this population, New York City can reduce the impact of this barrier.

People with less than a high school education were the third barrier. These citizens might have limited awareness of available resources and programs, and may have lower literacy levels (e.g., limited reading and writing skills). Increased disaster preparedness has been linked to higher levels of education^40^. New York City can tackle this by hosting more community and outreach events that give people the skills to execute preventative measures^41^.

The fourth identified barrier for New York City was traffic proximity and volume, which has long been recognized as a common impediment to effective EMS operations. Transportation infrastructure is vital to response efforts and can be heavily affected by disasters such as hurricanes^42^. It is best for the city to attempt to control this persistent issue by implementing strategies that can mitigate components of the transportation network, such as contraflow, if the solution is intended to be large in scale, or administer necessary lane closures to clear critical flow paths^43^.

It is important to note that many of the efforts to address all these identified barriers involve community participation, financial resources, legislative cooperation, and trained volunteers or workers. However, those involved in emergency management are often put in stress-induced scenarios, exposed to traumas, and are at risk for anxiety, depression, and post-traumatic stress disorder from the situations they must evaluate and make critical decisions on ^44^. This can lead to high turnover in volunteerism, staffing, and the lack of proper training and preparation^24^. Any effort to eliminate response disparities must consider these realities. We aim to initiate a critical dialog, emphasizing that addressing these matters equitably requires substantial efforts to transform a deeply ingrained system that may be plagued by systemic injustices.

Despite successfully rejecting the null hypotheses and obtaining statistically significant results, our study is not without its limitations. One limitation of this study is that it is difficult to generalize the findings of the present study to other areas. The case study to test our hypotheses was specifically on New York City’s reported time and its dispatch system. In general, with EMS times, the exact magnitude of and barriers to response will naturally fluctuate. This is partly due to the fact that communication systems vary from area to area. Therefore, it is important to consider the context and variations in applying these study findings to other EMS systems. However, the process and methods of our study are replicable in that future studies can adapt our methods to test these hypotheses in other cities.

Our study did not analyze at the individual level, but rather at the county level, which may overlook variations within the county (e.g., booming versus declining areas in the same borough). Thus, observed differences in EMS response should be interpreted as reflecting a combination of operational, geographic, infrastructural, and sociodemographic conditions across boroughs, rather than broad sociodemographic disparities alone. Spatial autocorrelation should also be further explored, as neighboring areas can share similar characteristics or response patterns. Additionally, recognizing that people are not "one size fits all," it is expected that there will be outliers regarding the disparities observed. An example could be tourists or those visiting who are unfamiliar with the emergency management system in New York City, and, in turn, they might experience delays or slower times due to miscommunications or lack of knowledge of their surroundings.

Furthermore, the barriers analysis was conducted using the most relevant available data, which corresponded to the year 2020. This year coincided with the global COVID-19 pandemic, which substantially disrupted infrastructure and public service systems related to EMS operations, including hospitals, transportation networks, travel behavior, and overall system demand. Studies have shown that COVID-19 significantly affected transportation usage and mobility patterns in major metropolitan areas such as New York City, influencing both disease spread and common transit behaviors^45^. As a result, some observed relationships may reflect conditions specific to the pandemic period rather than more stable long-term structural patterns. As additional post-pandemic data becomes available through future census releases and related datasets, future studies should compare these findings across time periods to evaluate the persistence of the observed relationships.

In the present study, demographics such as race and ethnicity were not included or considered as a barrier to response. This is because such characteristics are reported to be unknown before responder arrival^3^. However, racial disparities and systemic inequities exist in society^30,46^. While race variables are excluded from the de-identified dataset (i.e., no patient demographic information), geographic data can serve as a proxy for race, which could lead to racial discrimination^47^. Therefore, future work should explore this data at a more granular level and investigate relationships between response, socioeconomic status, and racial/ethnic disparities. Another future work is to compare rural versus urban areas regarding EMS response time disparities. The publicly available data were for a major metropolitan city in the U.S. However, assessing whether our hypotheses are still supported in a more rural area could determine if the present study method is replicable and pertinent in non-urban areas. Lastly, a future work that could stem from the present study would be to determine which particular call types experience this response time disparity. The dataset used in the present study from FDNY reported 169 unique call types classified as the final call type category. These can be further assessed to see which call types are most at risk, especially during or after a natural disaster such as a hurricane.

Practical next steps stemming from these findings include policy and operational reforms aimed at strengthening emergency medical programming and improving coordination across regional, municipal, and local emergency response systems. Building on prior research, such efforts should focus on addressing identified structural barriers and implementing targeted strategies to improve emergency response equity, accessibility, and system-wide efficiency^48^. Furthermore, for researchers who employ machine learning approaches to investigate problems, support decision-making, or develop recommendations, we strongly encourage the systematic incorporation of bias mitigation strategies for data collection, algorithm design, evaluation, and deployment to address potential computational inequities^49^. As artificial intelligence (AI) and machine learning become increasingly integrated into modern emergency management practice, addressing ethical considerations such as bias is essential for promoting the equitable and responsible use of these technologies, particularly within emergency response and management systems^50^. In parallel, developing governance frameworks that establish replicable and uniform standards for transparency, accountability, and oversight of AI-enabled decision-support systems will be critical. Such frameworks may be particularly valuable in emergency management, where protocols, resources, and operational practices can vary substantially across jurisdictions. Together, these approaches can support greater consistency in emergency management practices while helping ensure that computational tools contribute to improved service delivery and more equitable outcomes across communities.

## Methods

### Study design

**Fig. 3:**
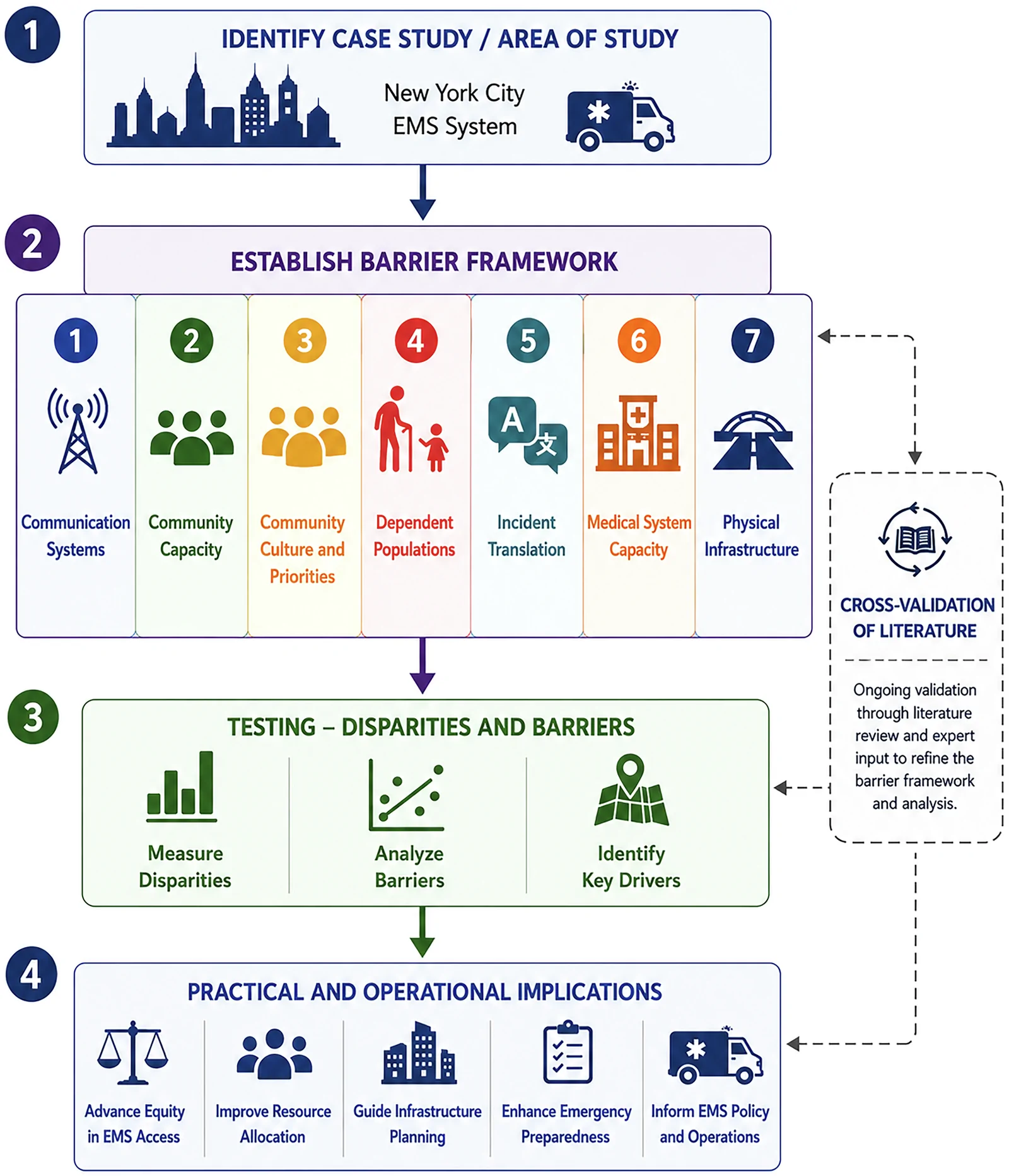
Conceptual diagram of the EMS barriers framework. The figure illustrates the study workflow, including case study identification, development of the EMS barrier framework through literature synthesis and cross-validation, quantitative testing of disparities and barriers, and interpretation of practical and operational implications related to emergency response equity and planning.

Figure 3 outlines the overall conceptual design of the study. The overall study process begins with identification of an EMS case study, followed by development of a barrier framework informed by emergency management literature and iterative cross-validation. The identified barriers were then quantitatively tested through disparity and barrier analyses to examine sociodemographic influences on EMS response times. Then, insights were subsequently interpreted in the context of broader practical and operational implications for emergency preparedness, infrastructure planning, resource allocation, and equity in EMS access as outlined in the “Results” section.

### Method overview

This section describes the methods used in the workflow in more detail. To test if there are significant disparities in time intervals related to EMS operations in the same area with shared dispatch systems, we first conducted an ANOVA on the five boroughs for dispatch time, travel time, and response time. All three time intervals were assessed to determine in which step EMS disparities may exist. We then followed with a post hoc test to further compare specific differences. Additionally, for industry standards, as defined earlier, response time was determined to be Dispatch Time + Travel Time, as outlined by the EMS data descriptions^17^ and aligning with the NFPA standard. NFPA 1710 standards for dispatch time should be completed within 90 s 90% of the time and within 120 s 99% of the time. For New York City, over the course of a decade, the percentage of values meeting the threshold of 90 s was 89.64%, and the percentage of values meeting the threshold of 120 s was 92.35%. For dispatch time, New York City closely aligns with national industry standards for EMS for both thresholds but still falls marginally below the desired benchmark. NFPA 1710 standards for travel time should be completed within 300 s. For New York City, over the course of a decade, the percentage of values meeting the threshold of 300 s was 30.17%. While NFPA does not specify a percentage requirement, New York City does not meet national industry standards for EMS in a significant portion of travel time occurrences.

The post hoc test selected was Games-Howell, as it accounts for unequal sample sizes and unequal variances, and is known to be conservative with Type I error rates (i.e., a reliable tool for determining statistical significance)^51^. After executing Games-Howell for dispatch time, travel time (see Supplementary Tables 2 and 3), and response time (see Table 2), we then further quantified the disparities seen for the overall EMS time interval into a single value by conducting a meta-analysis on the reported Hedges’ *g* effect sizes (see Eq. 1), which is an unbiased estimate for response time^36^.

1 $${\mathrm{Hedge}}{{\mathrm{s}}}^{{\prime} }{\mathrm{g}}=\frac{\bar{{{\mathrm{y}}}_{1}}-\bar{{{\mathrm{y}}}_{2}}}{{{\mathrm{s}}}_{{\mathrm{p}}}},$$

Where $\bar{{y}_{1}},\bar{{y}_{2}}$ are the means of sample 1 and sample 2, and ${s}_{p}$ is the pooled standard deviation

To ensure the different effect sizes initially calculated were all represented in the summary estimate, the random-effects model was used to combine each respective effect size for response time^52^. The weights were calculated by the inverse of the variance, with the variance being the square of the standard errors reported (for within-study), and the weighted mean across all studies was used to obtain the combined value^52^. The common approach of weighted least squares was used to calculate the weighted mean^53^. The random-effects model is also a way to reduce bias in the summary estimate. We calculated this to interpret the magnitude and significance of the disparities found in the total emergency response time (i.e., dispatch time and travel time).

After demonstrating disparities in an area that uses the same dispatch system designed to provide standardized treatment, we utilized regression to determine which outlined barriers most contribute to the response phase inequities established. Linear regression was determined to be the best model for our problem and dataset after considering data quantity (i.e., sample size) and an important aim of the present study (i.e., to predict and identify relationships between the dependent and independent variables). This dataset was for the year 2020, to correspond with available data, and consisted of 1,363,382 data points representing the response times reported. The barriers examined, based on literature, are as follows in Table 1.

The dependent variable for the test is the reported response times, and the independent variables are the barriers or variables outlined in Table 2 and described above. For independent variables, the dataset had 95 unique samples (i.e., 5 samples per variable), each representing a specific county's data in 2020. The sample size of 5 samples per variable is sufficient for linear regression to achieve proper estimation of statistical outputs such as coefficients and standard error^54^. Each data point associated with its respective county was merged with the appropriate unique reported response time throughout the dataset. This ensured that the dependent variable (i.e., response times) aligned appropriately with its specific area. Furthermore, it's important to note that while we have 5 unique observations per independent variable, there are significantly more unique values for the dependent variable, representing the diverse range of response times captured in the dataset. This allows for a comprehensive analysis of the relationship between the independent variables and the dependent variable, considering the variability in response times across the entire dataset while keeping location information consistent.

Next, we ran a multivariate analysis to see if there was multicollinearity between variables and found there was a significant degree of multicollinearity among the 19 variables. To handle the multicollinearity and achieve our objective to see which barrier is most statistically influential, the linear model of LASSO regression was performed to analyze which of the quantitative variables were truly most impactful to response time differences in New York City. LASSO regression was specifically used because it not only accounts for multicollinearity, but also overfitting and automatic variable selection of relevant predictors^55^.

Equation 2 captures how the coefficient estimates for the LASSO regression are defined^56^. The barriers data was combined into a single spreadsheet, then merged with the FDNY EMS Incident Dispatch Data on each dataset’s equivalent “county” column. Using JMP statistical software, the remainder of the analysis was executed. Z-score standardization was performed on the barriers to make the data unitless and comparable between each independent variable. LASSO regression was performed, splitting the data into training, validation, and testing sets in a 70:20:10 ratio, and then again using cross-validation to further detect potential overfitting. The LASSO regression performed to find the barriers was done with 2020 EMS data. In addition to the availability and recency of data in the year 2020, the decision to utilize a single year was further influenced by an observation of the density of the response times for the counties over time. The distribution patterns across all 10 years appeared proportionately consistent and comparable; therefore, using the most recent year could still capture the underlying disparity over time while maintaining the relevance of using the latest available data.

2 $${\hat{\beta }}^{\mathrm{lasso}}={\mathrm{argmin}}_{\beta }\left\{\frac{1}{2}\mathop{\sum }\nolimits_{{\mathrm{i}}=1}^{\mathrm{N}}-{\mathrm{LogLikelihood}}\left(\beta {\mathrm{;}}{\mathrm{y}}_{\mathrm{i}}\right)+\lambda \mathop{\sum }\nolimits_{{\mathrm{j}}=1}^{\mathrm{p}}\left|{\beta }_{\mathrm{j}}\right|\right\},$$

Where $\mathop{\sum}\nolimits_{{\mathrm{j}}=1}^{{\mathrm{p}}}\left|{\rm{\beta}}_{{\mathrm{j}}}\right|$ is the ${{\mathrm{l}}}_{1}$ penalty, λ is the tuning parameter, *N* is the number of rows, and *p* is the number of variables.

By performing the aforementioned statistical analyses, this study achieved its two key objectives: it established and quantified disparities in general emergency response times across various call types and severity levels, and it identified and tested potential barriers (i.e., pre-existing conditions) contributing to these disparities.

The overall analysis performed with these methods in the present study exposes inequities with EMS response times in a major metropolitan city whose EMS activities are operated by the same system (i.e., executed with the same operational systems). This study challenges the notion that providing uniform treatment to all individuals through EMS operations results in equal (i.e., fair and unbiased) service for all in response times. It highlights the potential presence of underlying societal issues that significantly impact equitable response times and emphasizes the importance of acknowledging and addressing these factors. Future policy interventions should focus more on equitable resource allocation, targeted infrastructure investments, and proactive strategies to enhance community resilience in the most socially susceptible areas. Addressing these disparities is essential to ensuring that all communities receive timely and effective emergency response services, particularly those at the highest risk.

## Supplementary information

Supplementary Materials

## Data Availability

The datasets generated and/or analyzed during the current study were obtained from publicly available data sources. Information on the original data sources and websites used to obtain the data is provided in Table 1. All datasets generated and/or analyzed during the current study are no longer publicly available due to changes in the availability of the original data sources following completion of the study, including the discontinuation or modification of some source repositories, but are available from the corresponding author upon reasonable request.
