# Supplementary material for: Inequities in urban emergency medical services
Source: Npj Health Syst. 2026 Sep 14;3:92. doi: 10.1038/s44401-026-00148-2 (PMC13575164; doi:10.1038/s44401-026-00148-2)
Supplement: Supplementary file 1 [file 44401_2026_148_MOESM1_ESM.pdf]

Supplementary Information

Supplementary Table 1. Key terms from the data set and NFPA 1710 that establish thresholds for EMS performance objectives[1,2].

**Time Interval**	**FDNY Terms for Time Interval**	**FDNY Definitions**	**NFPA 1710 Equivalent Terms for Time Interval**	**NFPA 1710 Equivalent Term(s) Definition**
Dispatch Time	Incident datetime<br>First assignment datetime	"The date and time the incident was created in the dispatch system."<br>"The date and time the first unit is assigned."	Alarm Processing Time	"The time interval from when the alarm is acknowledged at the communication center until response information begins to be transmitted via voice or electronic means to emergency response facilities (ERFs) and emergency response units (ERUs)."
Travel Time	First assignment datetime	"The date and time the first unit is assigned."	Turnout Time	"The time interval that begins when the emergency response facilities (ERFs) and emergency response units (ERUs) notification process begins by either an audible alarm or visual annunciation or both and ends at the beginning point of travel time."
	First on scene datetime	"The date and time the first unit signals that it has arrived at the location of the incident."	Travel Time	"The time interval that begins when a unit is en route to the emergency incident and ends when the unit arrives at the scene."
Response Time (Dispatch Time + Travel Time)	Incident datetime	"The date and time the incident was created in the dispatch system."	Alarm Processing Time	"The time interval from when the alarm is acknowledged at the communication center until response information begins to be transmitted via voice or electronic means to emergency response facilities (ERFs) and emergency response units (ERUs)."
	First on scene datetime	"The date and time the first unit signals that it has arrived at the location of the incident."	Turnout Time	"The time interval that begins when the emergency response facilities (ERFs) and emergency response units (ERUs) notification process begins by either an audible alarm or visual annunciation or both and ends at the beginning point of travel time."
			Travel Time	"The time interval that begins when a unit is en route to the emergency incident and ends when the unit arrives at the scene."

The "FDNY Terms for Time Interval" displays the terms that encompass the entire chronology of each time interval as defined by FDNY, while the "NFPA 1710 Equivalent Terms for Time Interval" presents the comparable terms that encompass the entire chronology of each time interval as seen in NFPA 1710. It should be noted that the definitions in the "FDNY Terms for Time Interval" column may not align precisely with the adjacent definitions in the "NFPA 1710 Equivalent Terms for Time Interval" column as it relates to the part of the time interval the individual definition is covering. That is, the terms are simply listed in each column in their appropriate sequences and time interval categories. All FDNY and NFPA 1710 terms listed per time interval span the same timeline and represent the respective time intervals holistically.

1. Fire Department of New York City (FDNY). (2016). *EMS Incident Dispatch Data: NYC open data*. EMS Incident Dispatch Data | NYC Open Data. https://data.cityofnewyork.us/Public-Safety/EMS-Incident-Dispatch-Data/76xm-jjuj

2. NFPA 1710. (2020). NFPA 1710: Standard for the Organization and Deployment of Fire Suppression Operations, Emergency Medical Operations, and Special Operations to the Public by Career Fire Departments, 2020 Edition. In *NFPA National Fire Codes Online*. Retrieved from http://codesonline.nfpa.org

Supplementary Table 2. Games-Howell Post Hoc Test for Dispatch Time.

Group 1	Group 2	Mean Group 1	Mean Group 2	Difference	p-value	Hedges	Reject Null Hypothesis 1?
BRONX	BROOKLYN	124.4234	72.07665	52.34679	<0.01	0.094213	TRUE
BRONX	MANHATTAN	124.4234	114.977	9.446473	<0.01	0.015503	TRUE
BRONX	QUEENS	124.4234	55.6457	68.77773	<0.01	0.12177	TRUE
BRONX	RICHMOND / STATEN ISLAND	124.4234	40.91024	83.51319	<0.01	0.143201	TRUE
BROOKLYN	MANHATTAN	72.07665	114.977	-42.9003	<0.01	-0.07658	TRUE
BROOKLYN	QUEENS	72.07665	55.6457	16.43095	<0.01	0.032181	TRUE
BROOKLYN	RICHMOND / STATEN ISLAND	72.07665	40.91024	31.16641	<0.01	0.062174	TRUE
MANHATTAN	QUEENS	114.977	55.6457	59.33126	<0.01	0.104102	TRUE
MANHATTAN	RICHMOND / STATEN ISLAND	114.977	40.91024	74.06672	<0.01	0.125594	TRUE
QUEENS	RICHMOND / STATEN ISLAND	55.6457	40.91024	14.73546	<0.01	0.029619	TRUE

Supplementary Table 3. Games-Howell Post Hoc Test for Travel Time.

Group 1	Group 2	Mean Group 1	Mean Group 2	Difference	p-value	Hedges	Reject Null Hypothesis 1?
BRONX	BROOKLYN	490.5507	472.2201	18.33063	<0.01	0.048278	TRUE
BRONX	MANHATTAN	490.5507	469.7831	20.76769	<0.01	0.051079	TRUE
BRONX	QUEENS	490.5507	453.4614	37.08931	<0.01	0.100339	TRUE
BRONX	RICHMOND / STATEN ISLAND	490.5507	415.7428	74.80796	<0.01	0.190282	TRUE
BROOKLYN	MANHATTAN	472.2201	469.7831	2.437065	<0.01	0.006336	TRUE
BROOKLYN	QUEENS	472.2201	453.4614	18.75868	<0.01	0.054036	TRUE
BROOKLYN	RICHMOND / STATEN ISLAND	472.2201	415.7428	56.47733	<0.01	0.157848	TRUE
MANHATTAN	QUEENS	469.7831	453.4614	16.32162	<0.01	0.043399	TRUE
MANHATTAN	RICHMOND / STATEN ISLAND	469.7831	415.7428	54.04027	<0.01	0.134691	TRUE
QUEENS	RICHMOND / STATEN ISLAND	453.4614	415.7428	37.71865	<0.01	0.114746	TRUE
